# Face Identity Recognition and the Social Difficulties Component of the Autism-Like Phenotype: Evidence for Phenotypic and Genetic Links

**DOI:** 10.1007/s10803-018-3539-4

**Published:** 2018-03-16

**Authors:** Gary J. Lewis, Nicolas G. Shakeshaft, Robert Plomin

**Affiliations:** 1 Department of Psychology, Royal Holloway, University of London, Egham, Surrey TW20 0EX UK; 2 King’s College London, MRC Social, Genetic and Developmental Psychiatry Centre, Institute of Psychiatry, De Crespigny Park, London, SE5 8AF UK

**Keywords:** Face memory, Object memory, Genetics, Twins, Autism-like traits

## Abstract

Autism spectrum disorder (ASD) and autism-like traits are associated with deficits in face memory ability, although it is not yet clear whether this deficit reflects a specific aspect of the ASD/autism-like phenotype. We addressed this issue using a neurotypical sample of adolescent twins (N_complete pairs_ = 782) drawn from the Twins Early Development Study who were assessed on face and object memory performance alongside two core aspects of autism-like traits: (i) difficulties with social behavior/interactions, and (ii) attention to detail. We observed a negative association between face memory ability and difficulties with social behavior/interactions. This association reflected an overlapping genetic etiology: heritable influences acting on face memory ability are associated with the social difficulties aspects of autism-like traits.

## Introduction

Autism spectrum disorder (ASD) is a neurodevelopmental disorder characterized by, among other features, socio-cognitive deficits (American Psychiatric Association 2013), such as for emotion expression recognition (Harms et al. 2010; Lozier et al. 2014). However, it is becoming clear that ASD and autism-like traits in the general population are also related to deficits in face memory (Weigelt et al. 2012). Nonetheless, a number of important questions have yet to be answered in this domain. Firstly, ASD and autism-like traits are now widely acknowledged to reflect a range of features (e.g. social interaction difficulties, fixation on patterns/details) which are largely unrelated (Hoekstra et al. 2008; Ronald et al. 2006): which of these underlying components relates to face memory? Secondly, are these associations sex-dependent? Thirdly, are face memory deficits in ASD and autism-like traits reflective of face-specific processes, or broader object-recognition processes? Fourthly, while the underlying etiologies of face memory and ASD/autism-like traits are both known to reflect genetic and environmental factors (Ronald and Hoekstra 2011; Shakeshaft and Plomin 2015; Wilmer et al. 2010), how do these sources of influence contribute to the established phenotypic associations between face memory and ASD or autism-like traits? Here we address these issues specifically focusing on autism-like traits assessed in a general population sample of adolescent twins. We next provide an overview of these issues before moving to an outline of our core analyses.

### Autism Spectrum Disorder, Autism-Like Traits, and Face Memory: An Overview

Although most face processing work concerning ASD has focused on characterizing deficits in theory of mind/mentalizing (Baron-Cohen 2000) and emotion recognition ability (Harms et al. 2010)—indeed, such deficits are diagnostic criteria for ASD (American Psychiatric Association 2013)—there is a growing literature examining face identity processing ability. In summary, ASD and autism-like traits do not appear to be associated with qualitative differences in face identity processing in general terms, nor with face identity perception (particularly when there is no delay between presentation of stimuli) (Weigelt et al. 2012).

However, several studies have highlighted quantitative deficits in face identity recognition ability, and particularly when memory processes are engaged (i.e. a delay between stimuli presentations: see Weigelt et al. 2012, for further details). For example, de Gelder et al. (1991) observed that children with an ASD diagnosis performed significantly poorer than a control group on a two-alternative forced-choice old–new face recognition test. Similarly, Boucher and Lewis (1992) reported that adolescents with an ASD diagnosis performed significantly worse than a control group on face memory but not house memory. Converging evidence of a face memory deficit in ASD has also been reported using standardized tests of face recognition ability, including the Cambridge Face Memory test (Kirchner et al. 2011) and the Warrington Recognition Memory Test for Faces (Blair et al. 2002).

While face memory deficits appear to be a fairly robust feature of the ASD phenotype there are a number of important outstanding questions in the field that require attention. Firstly, a growing body of work indicates that the social and cognitive difficulties associated with ASD are not manifestations of a unitary deficit. For example, work using assessments drawn from the Autism Quotient (AQ) instrument (Baron-Cohen et al. 2001) has identified two overarching and broadly distinct factors: a social interaction difficulties factor and an attention to details/numbers and patterns factor (Hoekstra et al. 2008, 2011; Kuenssberg et al. 2014). Twin studies have also highlighted the distinct genetic etiologies underlying the social and cognitive deficits reflective of ASD (Ronald et al. 2006). Collectively these findings highlight that ASD traits are heterogeneous in nature. As such, assessment at the aggregate level may fail to observe important associations that exist at the component level.

Of the few studies to date to have addressed whether face memory is differentially related to these aspects of autism-like traits, results have been mixed. For example, some work has reported a negative association between the social interaction difficulties aspect and face memory performance (Sasson et al. 2013). However, others have observed this association only with males (Rhodes et al. 2013) or females (Davis et al. 2017), respectively. With regards to the attention to detail component, some evidence suggests a positive association with face memory (Rhodes et al. 2013), although other work failed to observe this association (Davis et al. 2017: note, in this study an indirect effect of attention to detail on face memory was observed). Of note, in each of these studies sample sizes were relatively small (N < 250) and underpowered to reliably detect what are likely to be modest associations.

Secondly, the majority of studies in the field have examined face memory performance without the inclusion of a comparable object memory task. As such, it is unclear whether the reported face memory deficits reflect face-specific difficulties or challenges with object memory more broadly. Indeed, while some studies have noted a specific impairment for face memory (Boucher and Lewis 1992; Hauck et al. 1998; McPartland et al. 2011), several other studies have reported that, while face memory deficits were present in ASD, deficits in non-face memory performance were also apparent (Blair et al. 2002; O’Hearn et al. 2010; Williams et al. 2005).

Thirdly, while both face memory and ASD (both clinically-speaking and in general population terms) are underpinned by genetic and environmental influences (Ronald and Hoekstra 2011; Shakeshaft and Plomin 2015; Wilmer et al. 2010), it is unknown how these underlying factors contribute to the observed phenotypic links.

### The Current Study

The current study seeks to address these issues using a large sample of neurotypical late-adolescence monozygotic and dizygotic twins drawn from the Twins Early Development Study (TEDS: Haworth et al. 2013) who completed a standardized test of face recognition ability—the Cambridge Face Memory Test (CFMT: Duchaine and Nakayama 2006) and an analogous test of object-recognition ability—the Cambridge Car Memory Test (CCMT: Dennett et al. 2012). To assess autism-like traits we used a measure of the Autism Quotient, and in particular the two underlying higher factors—‘social behavior/interaction difficulties’ and ‘attention to detail/numbers and patterns’ (Hoekstra et al. 2011). We examined the relationship between these components of autism-like traits and face memory ability (controlling for object-recognition ability), as well as the underlying genetic and environmental etiology of the observed phenotypic links.

## Method

### Participants

The current study sample was drawn from two assessment waves—age 16 (when autism-like traits were assessed) and age 18 (when face and object memory were assessed)—in the Twins Early Development Study (TEDS). TEDS is an ongoing longitudinal study following monozygotic (MZ) and dizygotic (DZ) twins born in England and Wales between 1994 and 1996 (Haworth et al. 2013) and is representative of the UK population (Kovas et al. 2007). Ethical approval was provided by the King’s College London ethics committee (reference: 05/Q0706/228), and informed written consent was received for all participants. Zygosity was assessed at age 18 months (and again at age 3 years) through a parent questionnaire of physical similarity, which has been shown to be over 95% accurate when compared to DNA testing (Price et al. 2000). For cases where zygosity was unclear from this questionnaire, DNA testing was conducted (the zygosity determination consisted of a multiplex PCR assay containing up to 16 highly polymorphic microsatellite markers - the numbers of alleles for each marker varies between 7 and 17. The specific alleles and genotypes for each twin are compared to determine MZ or DZ status).

The current sample reflected a pseudo-randomly selected subset of the broader cohort (i.e. not all twins were recruited into the face memory study) and consisted of the following number of complete twin pairs: MZ pairs: N = 307; DZ pairs: N = 471 (same-sex: N = 256; opposite-sex: N = 215). Ninety-three% of participants were ‘White’, with the remainder being ‘Other’ (broken down by zygosity: MZ = 93% ‘White’; DZ same-sex = 94% ‘White’; DZ opposite-sex = 94% ‘White’). The current sample was 60% female and 40% male.

### Measures

Cambridge Face Memory Test (CFMT) (Duchaine and Nakayama 2006) requires participants to memorize six male Caucasian faces, each from three images with the face presented in different orientations. The faces are shown with neutral expressions and any distinguishing blemishes or hair and clothing are removed or cropped. In the test phases the target face is presented alongside two distractors. Trials fall into three distinct phases, the first following immediately after the memorization of each face (three trials for each, identifying that face among distractors), the second being a series of 30 trials in which the target can be any of the six memorized faces, and the third a series of 24 trials (again with any the memorized targets) using impoverished images degraded with Gaussian noise. Correct responses are summed with a maximum total score of 72.

Cambridge Car Memory Test (CCMT) (Dennett et al. 2012) was developed as a complement to the CFMT in order to provide a non-social matched comparison test. The stimuli are computer-generated 3D images of cars (which are life-like but not identifiable as a particular manufacturer or model) presented in various orientations. The procedure for memorization, testing, and scoring is identical to that for the CFMT.

Autism-like traits were assessed with parents completing the AQ-Short (Hoekstra et al. 2011), a 28-item instrument that assesses five domains of autism-like traits: ‘social skills’, ‘routine’, ‘switching’, ‘imagination’, and ‘attention to detail/numbers and patterns’. Some debate exists on the optimal factor solution of the AQ instruments (long-form and short-form versions: e.g. see Kloosterman et al. 2011; Stewart and Austin 2009), although the majority of confirmatory factor analyses have provided support for a model positing two higher-order factors (Hoekstra et al. 2008, 2011; Kuenssberg et al. 2014). As such, we adopted this approach in the current study.

The above work has noted that the first four of the AQ domains reflect a higher-order domain of ‘social behavior/interactions’ (Hoekstra et al. 2011). As such, we used this domain (denoted herein as AQ-social—constructed as the sum of the relevant sub-scales: 23 items in total)—alongside attention to detail/numbers and patterns (denoted herein as AQ-detail: 5 items in total) as our dependent variables of interest. Example items for the AQ-social measure were: “Finds social situations easy” (reverse scored); “Finds it hard to make new friends”; “Can easily keep track of several different people’s conversations” (reverse scored). Example items for the AQ-detail measure were: “Fascinated by dates”; “Notices patterns in things all the time”.

We also assessed self-report using an abridged version of the AQ-Short. Here AQ-social was constructed from all 7 items from the social skills domain and 1 item from the routine domain (“New situations make me anxious”), and all 5 items from the attention to detail/numbers and patterns domain). The self-report version of the test was shortened for pragmatic reasons: that is, so as not to overwhelm the adolescent participants with an overly long test battery. The items were chosen to best reflect the underlying two factor model detailed above (Hoekstra et al. 2008, 2011; Kuenssberg et al. 2014).

The parent- and self-report AQ-social and the self-report AQ-detail measures were approximately normally distributed (i.e. skew < 0.82, kurtosis < 0.61). The parent-rated AQ-detail measure showed similar skew and kurtosis values, but a degree of left-censoring was evident. Hoekstra et al. (2011) recommended that a total scale cut-off of > 65 can be useful to identify individuals for whom further clinical assessment might be appropriate. In the current sample this amounted to 9% of participants.

### Twin Analyses

Correlations between twins differing in their degrees of genetic-relatedness (i.e. MZ and DZ twins) are indicative with regards to the relative magnitudes of genetic and environmental effects (Knopik et al. 2017). The presence of genetic effects is inferred if the correlation between MZ twins is larger than the correlation for DZ twins. The presence of shared-environmental effects is inferred if the correlation for the DZ twins is larger than half the magnitude of the correlation for the MZ twins. Finally, nonshared-environmental effects are inferred if the correlation for the MZ twins is less than one, and so this variance component also contains measurement error. In line with standard practice, we used formal model-fitting of variance–covariance matrices for the twin data.

**Fig. 1:**
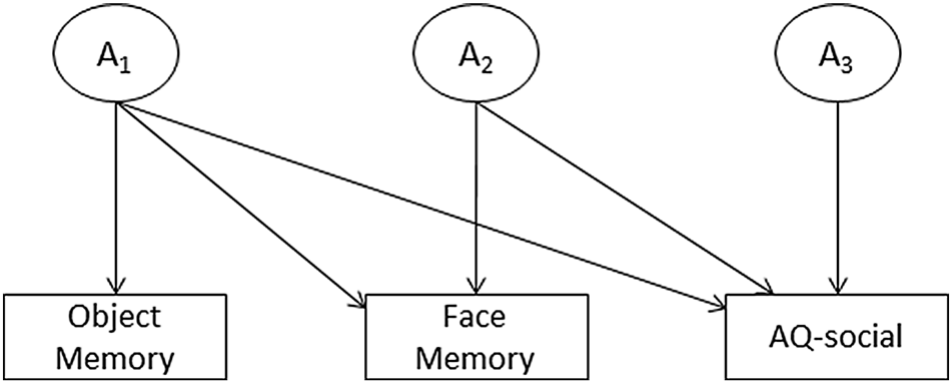
Schematic of the trivariate Cholesky decomposition for additive genetic effects. *Object memory* Cambridge Car Memory Test, *Face memory* Cambridge Face Memory Test, *A* additive genetic influences; shared-(C) and nonshared-environmental (E) influences are also modelled—and in the identical form as detailed for the genetic influences—but are omitted here for visual clarity

In the current study, multivariate twin analyses were central to our tests: We sought to estimate the extent to which genetic and environmental effects underlying autism-like traits are associated with face memory and object memory performance. To perform this analysis, we used the Cholesky decomposition. The Cholesky decomposition specifies as many factors as there are variables for each source of variance, with each subsequent factor having one fewer pathway than the preceding factor (see Fig. 1: Neale and Maes 2004). In other words, for additive genetic effects (A) the first latent factor loads on all of the n measured variables: The subsequent latent factors load on n − 1, n − 2 … n − i variables. In this way each factor accounts for as much of the remaining variance as possible, until the last factor accounts for just the residual variance in the last measured variable. This is repeated for the shared-environment factors (C) and nonshared-environmental factors (E). This design allows us to examine whether object memory performance has overlapping genetic and/or environmental influences with autism-like traits. Moreover, by entering face memory second in the order we can assess whether it has overlapping genetic and/or environmental influences with autism-like traits, independent of object memory. Twin models were fitted using full-information maximum-likelihood in OpenMx 2.6.9 (Boker et al. 2011, 2013) running within R 3.2.5 (R Development Core Team 2015).

## Results

### Phenotypic Analyses

Descriptive statistics for our study variables are presented in Table 1. Linear mixed effects models (family ID was modelled as a random effect) were used to examine the association between face/object memory and AQ-social/AQ-detail. Standardized parameter estimates are reported. We observed that face memory was a significant predictor of both self-report and parent-report AQ-social (β = − 0.06 [CI95%: − 0.11, − 0.01]; β = − 0.13 [CI95%: − 0.17, − 0.09], respectively). This association was still present when including object memory in the model (β = − 0.06 [CI95%: − 0.11, − 0.002]; β = − 0.14 [CI95%: − 0.19, − 0.10], respectively). We also observed that object memory was a significant predictor of both self-report and parent-report AQ-detail (β = 0.07 [CI95%: 0.02, 0.12]; β = 0.06 [CI95%: 0.02, 0.10], respectively), and this association was still present when including face memory in the model (β = 0.07 [CI95%: 0.01, 0.12]; β = 0.07 [CI95%: 0.03, 0.10], respectively). No other associations were observed. We next examined whether sex moderated these phenotypic associations by including an interaction term in our models; however, this interaction term was non-significant in all cases. Finally, sensitivity analyses removing participants who scored above the nominal cut-off (AQ-total score > 65) proposed by Hoekstra et al. (2011) showed virtually identical results, with all of the significant associations reported above still present.

**Table 1:** Descriptive statistics for the study variables

Measure	α	All M (SD)	MZm M (SD)	MZf M (SD)	DZm M (SD)	DZf M (SD)	DZOSm M (SD)	DZOSf M (SD)
AQ-social (self-report)	0.79	14.91 (4.09)	14.76 (3.99)	15.10 (4.26)	14.76 (3.85)	15.23 (4.34)	14.78 (3.79)	14.66 (4.11)
AQ_detail (self-report)	0.80	9.90 (3.50)	10.23 (3.51)	9.53 (3.41)	10.15 (3.55)	9.70 (3.51)	10.50 (3.60)	9.49 (3.33)
AQ-social (parent-report)	0.85	40.78 (9.72)	41.88 (9.57)	39.69 (9.31)	42.10 (9.99)	40.11 (9.49)	43.05 (10.65)	38.66 (8.71)
AQ_detail (parent-report)	0.84	9.35 (3.74)	9.50 (3.85)	8.86 (3.61)	9.47 (3.84)	9.24 (3.68)	10.18 (3.91)	9.05 (3.44)
Face memory	–	53.96 (9.75)	52.58 (9.88)	54.28 (9.42)	53.56 (10.19)	54.71 (9.87)	52.81 (9.87)	54.99 (9.40)
Object memory	–	50.04 (10.30)	54.03 (10.73)	47.51 (9.30)	53.98 (10.69)	47.27 (9.51)	53.01 (10.67)	48.33 (9.05)

*Face memory* Cambridge Face Memory Test, *Object memory* Cambridge Car Memory Test, *MZ* monozygotic, *DZ* dizygotic, *SS* same-sex, *OS* opposite-sex, *m* male, *f* female; one twin from each pair was drawn randomly for use in these analyses

### Twin Analyses

The pattern of twin correlations (see Table 2) indicated the presence of additive genetic influences for all phenotypes (i.e. MZ twin pairs were correlated more highly than DZ twin pairs), along with evidence for shared-environmental influences for parent-reported AQ-social and AQ-detail (i.e. DZ twin pair were correlated at more than half of the correlation for the MZ twin pairs). These descriptive patterns were confirmed by formal model fitting tests (see Table 2). For all variables, additive genetic and nonshared-environmental influences were significant. For parent-reported AQ-social and AQ-detail shared-environmental influences were also significant.

**Table 2:** Twin correlations and univariate analysis results

Measure	MZr	DZSSr	DZOSr	A	C	E
AQ-social (self-report)	0.57	0.22	0.15	0.56 [0.52–0.61]	0.00 [0.00–0.04]	0.44 [0.41–0.46]
AQ_detail (self-report)	0.44	0.19	0.13	0.42 [0.38–0.46]	0.00 [0.00–0.09]	0.58 [0.55–0.61]
AQ-social (parent-report)	0.88	0.54	0.47	0.71 [0.64–0.77]	0.18 [0.12–0.25]	0.12 [0.11–0.12]
AQ_detail (parent-report)	0.93	0.77	0.70	0.34 [0.30–0.37]	0.59 [0.55–0.64]	0.07 [0.06–0.07]
Face memory	0.62	0.29	0.16	0.62 [0.53–0.72]	0.00 [0.00–0.43]	0.37 [0.33–0.42]
Object memory	0.63	0.27	0.26	0.58 [0.49–0.67]	0.00 [0.00–0.11]	0.41 [0.36–0.46]

*Object memory* Cambridge Face Memory Test, *Face memory* Cambridge Car Memory Test, *MZ* monozygotic, *DZ* dizygotic, *SS* same-sex, *OS* opposite-sex; all correlations p < .01; *A* additive genetic effects, *C* shared-environment effects, *E* nonshared-environment effects; CI95% in square brackets

We next moved to our multivariate twin analyses. Here we focused on the parent-report AQ-social measure as the observed phenotypic association was larger and because it assessed the full social interactions difficulties domain (in contrast to the self-report AQ-social which only assessed a facet of the social domain). As detailed above we built a trivariate Cholesky decomposition with the following variables ordered from left to right: object memory, face memory, and AQ-social. As detailed above, this allowed us to assess the genetic and environmental links between face memory and AQ-social independently of any object memory associations with AQ-social.

We first examined shared-environment parameters. With the exception of AQ-social (0.42), all of these parameters were at, or close, to zero (i.e. ≤ 0.03). Removing these shared-environment parameters did not significantly worsen model fit (Δχ^2^ (5) = 0.07, p = .99). Next, to test whether object and face memory were genetically associated with AQ-social we examined each of the A and E paths shared between object memory and AQ-social, and face memory and AQ-social. These parameters correspond to A1 → AQ-social and A2 → AQ-social, respectively. In each case removing the path resulted in a significant worsening of model fit (Δχ^2^ (1) = 14.92, p = .0001; Δχ^2^ (1) = 7.70, p = .006), indicating a genetic link between these variables. Specifically, genetic influences that give rise to better object and face memory performance are associated with a lower level of social interaction difficulties. We performed the same tests for nonshared-environmental influences; here we found a link from E1 → AQ-social (Δχ^2^ (1) = 7.31, p = .007)—nonshared-environmental influences that give rise to better object memory performance are associated with higher levels of social interaction difficulties – but not for E2 → AQ-social (Δχ^2^ (1) = 0.08, p = .77).

**Fig. 2:**
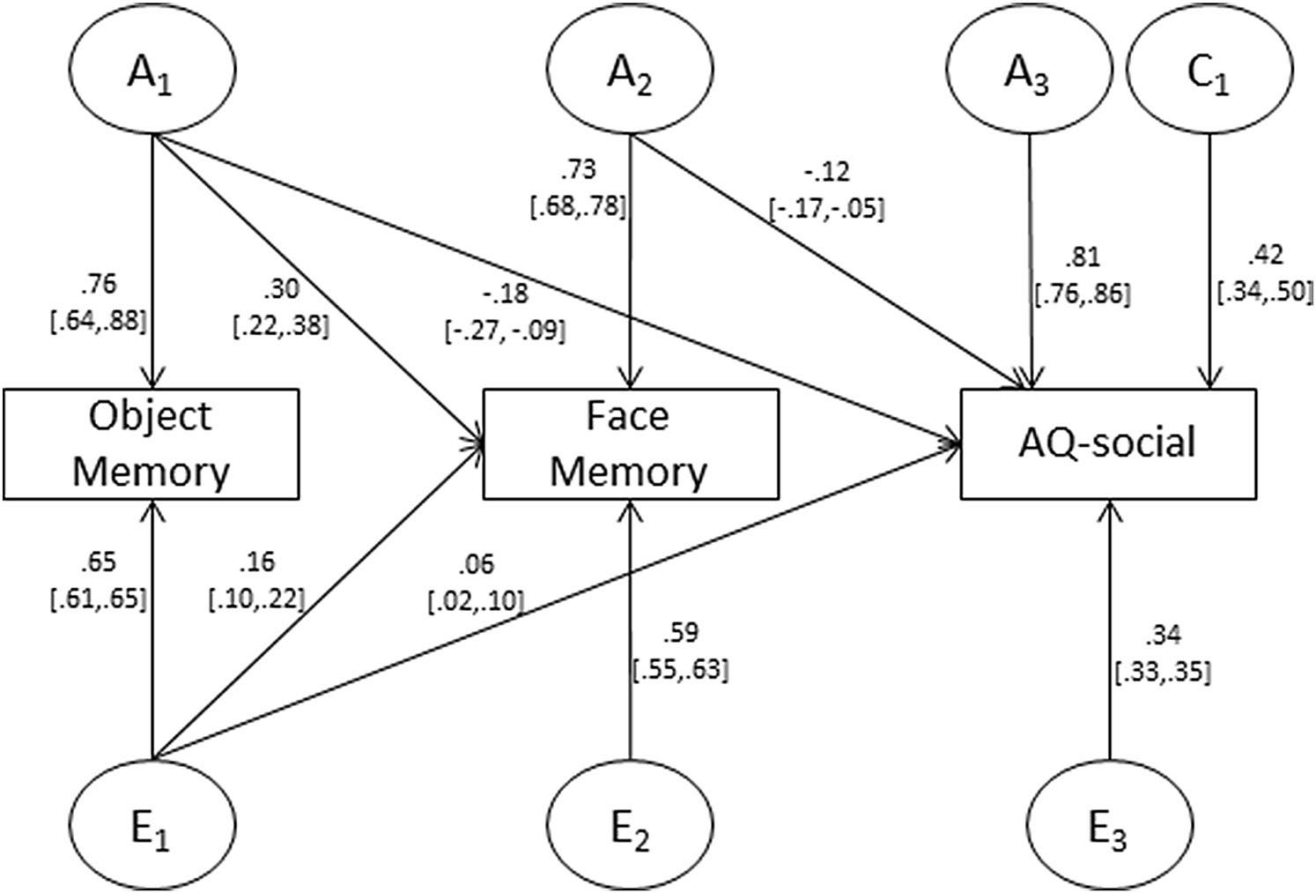
Reduced Cholesky output for object memory, face memory, and AQ-social. Values reflect standardized path coefficients; CI95% in square brackets; *Object memory* Cambridge Car Memory Test, *Face memory* Cambridge Face Memory Test, *A* additive genetic influences, *C* shared-environmental influences, *E* nonshared-environmental influences

The reduced Cholesky decomposition (i.e. with the non-significant paths removed) is detailed in Fig. 2. These findings collectively demonstrate that the phenotypic correlation between face memory and AQ-social—net of any links with object memory—was entirely attributable to genetic overlap (note, in the full Cholesky decomposition genetic factors accounted for 99% of the phenotypic overlap indicating this result was not the outcome of the reduced model).

## Discussion

The current study sought to answer the following questions: (1) what is the phenotypic relationship between two of the underlying components of autism-like traits—difficulties with social interactions and attention to detail/numbers and patterns—and face memory ability?; (2) are these associations sex-dependent?; (3) do face memory deficits in these autism-like traits reflect face-specific processes, or broader object-recognition processes?; and (4) does the association between face memory and autism-like traits reflect genetic and/or environmental factors?

Our findings highlight the following. Firstly, face memory ability showed a modest negative association with autism-like traits in our general population sample—specifically with the social interactions difficulties aspect, which is consistent with some recent work (Sasson et al. 2013; Davis et al. 2017; Rhodes et al. 2013). In addition, we observed that object memory had a modest positive phenotypic association with AQ-detail.

Secondly, in contrast to some recent accounts (Davis et al. 2017; Rhodes et al. 2013), this association was not sex-dependent. Given the relatively large sample available here, the failure to replicate the sex-limited findings may indicate sample-specific observations in prior work of this kind.

Thirdly, this association was independent of object memory ability, indicating that the face memory deficit link with autism-like traits is not reflective of a broader memory deficit. This observation provides important information in line with mixed findings concerning the specificity of the memory deficit (Blair et al. 2002; Boucher and Lewis 1992; Hauck et al. 1998; McPartland et al. 2011; O’Hearn et al. 2010; Williams et al. 2005). It is possible, however, that this association is not face-specific in kind, but instead reflects a broader social perception ability, perhaps extending across modalities (e.g. Lewis et al. 2016).

Fourthly, the phenotypic association between face memory and the social aspects of the autism phenotype reflects a common genetic etiology: in other words, the genetic factors that lead to enhanced face memory ability are associated with greater ability in social interactions. Of further note, genetic factors accounted for the entirety of the phenotypic correlation between face memory ability and AQ-social score.

As noted earlier, autism spectrum disorder and autism-like traits in the general population are often characterised as a unitary disorder (Ronald et al. 2006). These results further emphasize the importance of moving away from this conceptualization. Indeed, to better characterise the socio-cognitive deficits associated with autism spectrum disorder—both in clinical cases and in the autism-like traits seen in the general population, as exampled here—would appear to require addressing its distinct characteristics. Future work in this vein is thus recommended.

While we observed genetic and nonshared-environmental associations between face memory and difficulties with social interactions, with the current study design we cannot draw conclusions with regards to the direction of causality underpinning this relationship. For example it is conceivable that a lack of social motivation deleteriously impacts on the development of social cognition (Chevallier et al. 2012), or that autism spectrum disorder and autism-like traits are fundamentally characterized by impairment to socio-cognitive faculties that in turn lead to reduced interests in social interactions. Research assessing these phenotypes using an early-life longitudinal design will be required to answer these important questions. It is also conceivable that a third variable(s) might explain this relationship. Future work may thus also want to consider including a broader set of covariates than reported here.

Some limitations of the current study require mention: firstly, our sample consisted of adolescents who may still be in flux with regards to the development of the processes underpinning face memory performance (e.g. Susilo et al. 2013), although other recent work such as Fuhrmann et al. (2016) suggests little if any improvement in performance is observed beyond the age of 18, which was the age of testing in the current sample. Secondly, our measures of autism-like traits and object/face memory were taken at age 16 and age 18, respectively. The primary implication of this time lag will be the attenuation of the associations between our study variables. As such, the true covariance between the autism-like social traits and face memory might be larger than reported here. Thirdly, here we used the AQ-short measure to assess autism-like traits—and in particular, the two higher-order factors that have been identified in several studies (Hoekstra et al. 2008, 2011; Kuenssberg et al. 2014). However, some studies have reported alternative factor solutions (e.g. Kloosterman et al. 2011; Stewart and Austin 2009). As such, future work may wish to explore links between face memory and broader aspects of the autism-like phenotype. Fourthly, the AQ-detail measure was brief in scope. As such it is possible that the construct is not being assessed in its full sense. Future work might thus wish to use a broader instrument, perhaps trading off items from the longer AQ-social domain. Finally, the classical twin design is subject to a number of assumptions, such as the equal environments assumption (Knopik et al. 2017). As such, future research that can capitalize on additional family structures (e.g. the extended twin design, and ultimately DNA studies) in order to provide more assumption-free estimates would be valuable. Nonetheless, it is noteworthy that formal tests of the extent to which violations of the equal environments assumption are present have found little evidence for this potential source of bias (e.g. Kendler et al. 1993).

In summary, the current study observed phenotypic and genetic associations between face memory ability and the social difficulties aspect of the autism-like phenotype. Of note, these associations were independent of object recognition ability and so indicate that the links to autism-like traits, at least with regard to difficulties in social behaviour/interaction, are somewhat more focal in kind and may reflect face-specific memory impairment. As such, these findings help to further refine the socio-cognitive deficits associated with autism-like traits. Future research is recommended to establish the causal pathways of this association.
